# Early Predictive Value of the Glucose‐to‐Lymphocyte Ratio for the Occurrence of Stroke‐Associated Pneumonia

**DOI:** 10.1002/brb3.70404

**Published:** 2025-03-09

**Authors:** Fuqiang Zhou, Liju Ma, Min Li, Haijiang Li, Heying Yang, Ye Xu, Kuankuan Dang, Fengchen Gao, Haimei Sun

**Affiliations:** ^1^ Department of Neurology First Affiliated Hospital of Kunming Medical University Kunming Yunnan China; ^2^ Department of Yunnan Key Laboratory of Laboratory Medicine First Affiliated Hospital of Kunming Medical University Kunming China; ^3^ Department of Infection Management Office First Affiliated Hospital of Kunming Medical University Kunming China; ^4^ Department of Intensive Care Medicine Yan'an Hospital Affiliated to Kunming Medical University Kunming China; ^5^ Department of Operations Management First Affiliated Hospital of Kunming Medical University Kunming China; ^6^ School of Public Health Kunming Medical University Kunming China

**Keywords:** glucose‐to‐lymphocyte ratio (GLR), predictive value, stroke, stroke‐associated pneumonia (SAP)

## Abstract

**Background and Objective:**

Hyperglycemia and poststroke immunosuppression can lead to a decline in immune function, resulting in an increased incidence of infectious events. The relationship between the glucose‐to‐lymphocyte ratio (GLR), a novel indicator, and stroke‐associated pneumonia (SAP) remains unclear. The objective of this study is to investigate the early predictive value of the GLR in the context of SAP.

**Methods:**

A retrospective analysis was conducted on acute stroke patients admitted to the Department of Neurology at the First Affiliated Hospital of Kunming Medical University from 2017 to 2021. The dataset included demographic information, vascular risk factors, and laboratory test results. Logistic regression analysis was used to assess the correlation between the GLR and the incidence of SAP. The GLR was converted into a categorical variable for trend testing, and compared the predictive efficiency of GLR through the receiver operating characteristic (ROC) curve and Bonferroni correction analysis.

**Results:**

This study included 711 patients with acute stroke according to a 1:2 case–control ratio, with 237 (33.3%) in the SAP group and 474 (66.7%) in the Non‐SAP group. The baseline level of the GLR was significantly greater in the SAP group than in the Non‐SAP group (*p* < 0.001). After correction using multifactorial logistic regression analysis, GLR (OR: 1.182, 95% CI: 1.090–1.281, *p* < 0.001) was identified as an independent risk factor for SAP. When GLR was converted into a categorical variable, the risk of SAP in group Q3 was 3.210 times greater than that in group Q1, and the trend test yielded *p* < 0.001. The analysis of the ROC curve revealed that the area under the curve (AUC) for the GLR was 0.737, with a sensitivity of 70.0% and specificity of 67.1% at a cutoff value of 4.110. The predictive efficacy of the GLR for SAP patients was superior to that of either blood glucose or lymphocyte counts alone (*p* < 0.0167).

**Conclusions:**

An elevated GLR within 24 h of hospital admission following an acute stroke is an independent risk factor for SAP. The risk of SAP increases progressively with increasing GLR, suggesting that the GLR may have a certain early predictive value for the occurrence of SAP.

## Graphical abstract

Hyperglycemia and poststroke immunosuppression can lead to a decline in immune function, resulting in an increased incidence of infectious events. The relationship between the glucose‐to‐lymphocyte ratio (GLR) and stroke‐associated pneumonia (SAP) remains unclear, and it can reflect the body status of stroke patients in a more comprehensive way. This study investigates the early predictive value of the GLR in the context of SAP and statistically compares it with previously relevant indicators. The results suggest that an elevated GLR within 24 h of hospital admission following an acute stroke is an independent risk factor for SAP. The risk of SAP increases progressively with increasing GLR.

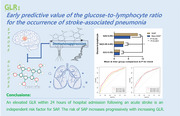

## 1 Background

Stroke represents a significant global public health burden, and data from the WHO 2019 Global Burden of Disease (GBD) study revealed that stroke ranks as the second leading cause of mortality and the third leading cause of death due to disability worldwide, with a staggering 70.0% increase in stroke events between 1990 and 2019. In 2019, the prevalence of stroke in China reached 28.76 million cases, marking a 147.5% increase from 1990 levels, with 3.94 million new cases reported that year (Collaborators GBDS 2021). Additionally, a 2023 study published in *JAMA* by Tu Wenjun et al. estimated that among individuals aged 40 years and older in China, there were 17.8 million prevalent cases, 3.4 million new cases, and 2.3 million deaths attributable to stroke in 2020 (Tu et al. 2023). Stroke‐related complications frequently result in poor prognosis, increased mortality, and escalated medical costs. Stroke‐associated pneumonia (SAP) is defined by the Pneumonia in Stroke Consensus (PISCES) group as new‐onset pneumonia within 7 days among nonmechanically ventilated stroke patients (Kishore et al. 2019). Available data indicate that the incidence of SAP varies between 7% and 38% (Ji et al. 2013; Smith et al. 2015; Teh et al. 2018; Yu et al. 2016), and the primary pathogenesis of the disease is associated with immunosuppression induced by stroke and aspiration resulting from impaired consciousness (Hannawi et al. 2013). Despite a gradual decrease in the incidence of stroke and improvements in treatment methods during the acute phase such as thrombolytic drugs and endovascular thrombectomy (EVT), the burden of stroke‐related complications remains substantial (Collaborators GBDS 2021; Tu et al. 2023). SAP serves as an important risk factor for poor prognosis among stroke patients, and its clinical manifestations are atypical during the early stages of disease progression. Consequently, investigating the early predictive indicators associated with SAP is highly important.

Abnormal elevation of blood glucose is recognized as one of the five major risk factors for stroke, contributing to 20.2% of total disability‐adjusted life years (DALYs) attributed to this condition (Feigin et al. 2022). A study conducted by Zonneveld et al. in 2017 demonstrated a significant association between hyperglycemia at admission and the development of poststroke infections such as pneumonia (Zonneveld et al. 2017). Research by Ji et al. in 2013 on the SAP scoring system (AIS‐APS) indicated that elevated blood glucose levels are correlated with the development of SAP (Ji et al. 2013). Mechanistic analyses indicate that dual immunosuppression induced by stroke and hyperglycemia is the primary cause of lymphocyte decline and increased susceptibility to pneumonia (Jafar et al. 2016; Nobs et al. 2023; Hoffmann et al. 2017; Shim and Wong 2016). Currently, several studies have explored the value of blood parameters such as the neutrophil‐to‐lymphocyte ratio (NLR) and systemic inflammatory response index (SIRI) for the prediction of SAP (Wang et al. 2023; Nam et al. 2018; Cui et al. 2023). The glucose‐to‐lymphocyte ratio (GLR) is a relatively novel indicator. In 2023, Yang et al. reported a linear relationship between increasing GLR and inpatient mortality rates among stroke patients (Yang et al. 2023); furthermore, Zhang et al. in 2022 established that the GLR serves as an independent predictor of inpatient mortality in patients with acute exacerbations of chronic obstructive pulmonary disease (AECOPD) (Hu et al. 2022). Additionally, previous research has shown that GLR is associated with conditions such as acute respiratory distress syndrome (ARDS), acute myocardial infarction (AMI), and certain neoplasms (Zhang and Zhang 2022; Liu and Hu 2023; Yang et al. 2022). However, to date, few studies have investigated the combination of admission blood glucose (ABG) and inflammatory factors for predicting the occurrence of SAP.

The GLR is a clinically accessible predictive indicator, which has been reported to be associated with AECOPD, all‐cause mortality of stroke, and other diseases. Previous studies have demonstrated a close relationship between high blood glucose levels and lymphocyte decline at stroke admission and the occurrence of SAP (Zonneveld et al. 2017; Hoffmann et al. 2017). The hyperglycemia caused by stress and other factors during a stroke may potentially provide hints for subsequent changes in immune function. We hypothesize that the GLR, which integrates both parameters, may more comprehensively and objectively reflect the immune status of patients at the time of stroke onset, and that the GLR may offer superior predictive value for SAP compared with single blood glucose or lymphocyte counts. However, to date, no research reports related to this content. This retrospective case study aims primarily to investigate whether the GLR upon admission in stroke patients has early predictive value for the development of SAP.

## 2 Methods

### 2.1 Study Subjects

The study population comprised acute stroke patients admitted to the Department of Neurology at the First Affiliated Hospital of Kunming Medical University between January 2017 and December 2021. The collection criteria were as follows: (i) Age of onset ≥18 years and clinical data are relatively complete; (ii) Meeting diagnostic criteria for acute stroke, acute stroke confirmed by head CT/MRI (Yew and Cheng 2015); (iii) Not receiving mechanical ventilation treatment after hospitalization; and (iv) No pulmonary infection or active infection within 2 weeks prior to admission. The exclusion criteria included the following: (i) Abnormal routine regional epidemiological testing upon admission; (ii) Use hormones or immunosuppressants within 2 weeks before admission; (iii) History of blood immune system diseases or malignancies; and (iv) Other conditions such as transient ischemic attacks (TIA), recent major trauma, or major surgical operations.

Stroke‐induced immune suppression and post‐stroke consciousness disorders, swallowing dysfunction leading to aspiration are considered the main pathogenesis of SAP (Hannawi et al. 2013). The diagnostic criteria for SAP were established by the SAP consensus group (PISCES), which consists of multidisciplinary experts from Europe and defines SAP as pneumonia occurring within 7 days in acute stroke patients who are not on mechanical ventilation (Kishore et al. 2019). The specific diagnostic criteria include the following: (i) Fever (temperature >38°C) or altered consciousness without an identifiable cause; (ii) Leukopenia or leukocytosis; (iii) New changes observed via chest imaging; and (iv) New respiratory secretions, dyspnea, or abnormal lung auscultation findings (Smith et al. 2015). Adopting a 1:2 paired case–control study, corresponding control matching is conducted based on the number of individual cases.

### 2.2 Data Collection

A retrospective analysis was conducted to collect demographic data (gender, age), vascular risk factors (atrial fibrillation, coronary heart disease, diabetes, hypertension, hyperlipidemia, smoking, drinking), history of stroke, history of proton pump inhibitor (PPI) medication, length of hospital stay, hospital survival status, stroke type, National Institutes of Health Stroke Scale (NIHSS) score, occurrence of SAP, and use of gastric tube. Relevant laboratory test results within 24 h of admission were also collected, including leukocyte count, absolute neutrophil count, absolute lymphocyte count, absolute monocyte count, erythrocyte count, hemoglobin level, platelet count, and admission blood glucose. The GLR index was calculated via the first admission blood glucose and absolute lymphocyte count within 24 h of admission = (admission blood glucose/absolute lymphocyte count). NLR = (absolute neutrophil count/absolute lymphocyte count) within 24 h of admission. SIRI = (absolute neutrophil count × absolute monocyte count/absolute lymphocyte count) within 24 h of admission.

### 2.3 Statistical Methods

Statistical analyses were conducted via SPSS version 27.0 and R version 4.4.1. The count data are expressed as percentages of cases [*n* (%)] and were compared between groups using the Chi‐square test. The measurement data conforming to normal distribution were expressed as the mean ± standard deviation, with group comparisons performed via *t*‐tests. Nonnormally distributed data are represented as median (interquartile range) [M(P25, P75)], and group comparisons were conducted via the Mann–Whitney *U* test. Multivariate logistic regression analysis was used to determine independent correlations between the GLR and SAP. GLR was categorized on the basis of tertiles for trend testing purposes, while the receiver operating characteristic (ROC) curve and paired comparative analysis with indicators were used to evaluate its predictive efficiency. A significance level of *p* < 0.05 was considered statistically significant. Pairwise comparison between the three groups was corrected using Bonferroni, and *p* < 0.0167 was considered statistically significant.

## 3 Results

### 3.1 General Characteristics of the Study Population

**FIGURE 1:**
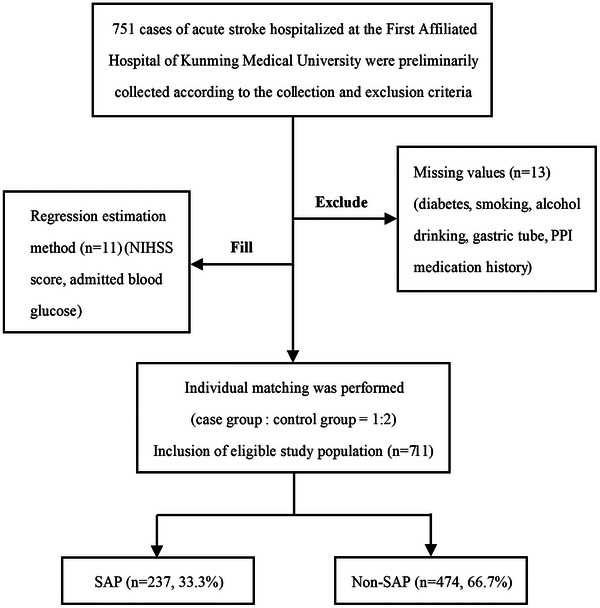
Flowchart of the data collection and processing procedure.

This study initially collected data from 751 cases. After data cleaning, a total of 711 cases were included according to individual matching (case group:control group = 1:2), with 237 cases (33.3%) in the case group and 474 cases (66.7%) in the control group, Figure 1 shows this process. In the final data of 711 cases, there were 86 patients (12.1%) with hemorrhagic stroke and 685 patients (87.9%) with ischemic stroke. The average age was 66 years old, and 450 patients (63.3%) were male. The baseline characteristics of the relevant variables were analyzed, revealing significantly greater levels in the SAP group than in the Non‐SAP group in terms of age, atrial fibrillation, history of PPI medication, and the levels of leukocytes, neutrophils, lymphocytes, monocytes, hemoglobin, admission blood glucose, GLR, NIHSS score, gastric tube use, in‐hospital mortality, and length of hospital stay. *p* < 0.05 was considered statistically significant (Table 1).

**TABLE 1:** Comparison of baseline characteristics between SAP group and Non‐SAP group.

Variables	SAP (*n* = 237)	Non‐SAP (*n* = 474)	*t*/*z*/*χ* ^2^	*p* value
Age, years [IQR]	69.00 (60.00, 79.00)	64.00 (54.00, 74.00)	−4.509	<0.001^*^
Gender, males, *n* (%)	146 (61.6%)	304 (64.1%)	0.436	0.509
**Vascular risk factors, *n* (%)**				
Hypertension	152 (64.1%)	307 (64.8%)	0.028	0.868
Hyperlipidemia	56 (23.6%)	127 (26.8%)	0.828	0.363
Coronary heart disease	24 (10.1%)	47 (9.9%)	0.008	0.930
Atrial fibrillation	27 (11.4%)	26 (5.5%)	7.992	0.005^*^
Diabetes	47 (19.8%)	98 (20.7%)	0.069	0.792
Smoking	93 (39.2%)	201 (42.4%)	0.652	0.419
Alcohol drinking	79 (33.3%)	154 (32.5%)	0.051	0.821
PPI medication history	197 (83.1%)	327 (69.0%)	16.286	<0.001^*^
**Laboratory results [IQR]**				
Leukocyte (×10^9^/L)	8.94 (6.71, 11.76)	7.06 (5.91, 8.63)	−7.178	<0.001^*^
Neutrophil (×10^9^/L)	7.15 (4.53, 9.39)	4.52 (3.53, 6.12)	−8.855	<0.001^*^
Lymphocyte (×10^9^/L)	1.24 (0.92, 1.71)	1.72 (1.36, 2.13)	−8.665	<0.001^*^
Monocyte (×10^9^/L)	0.51 (0.39, 0.71)	0.47 (0.37, 0.57)	−3.288	0.001^*^
Erythrocyte (×10^12^/L)	4.82 (4.42, 5.26)	4.87 (4.51, 5.25)	−1.333	0.183
Hemoglobin (g/L)	149.00 (135.00, 161.00)	151.00 (139.00, 163.00)	−1.956	0.049^*^
Platelet (×10^9^/L)	195.00 (157.00, 235.50)	202.50 (164.00, 245.00)	−1.452	0.147
Admitted blood glucose (mmol/L)	6.31 (5.46, 8.18)	5.39 (4.70, 6.50)	−7.345	<0.001^*^
Glucose‐to‐lymphocyte ratio (GLR)	5.16 (3.66, 8.91)	3.27 (2.49, 4.66)	−10.292	<0.001^*^
**Other variables**				
NIHSS score [IQR]	10.00 (4.00, 16.50)	3.00 (2.00, 6.00)	−11.761	<0.001^*^
Gastric tube, *n* (%)	130 (54.9%)	33 (6.9%)	205.080	<0.001^*^
Length of stay, >14 days, *n* (%)	71 (30.0%)	23 (4.8%)	86.800	<0.001^*^
In‐hospital mortality, *n* (%)	18 (9.5%)	20 (5.3%)	18.950	<0.001^*^

Abbreviations: IQR, interquartile range; NIHSS, National Institute of Health Stroke Scale.

^*^ *p* < 0.05.

### 3.2 Logistic Regression Analysis

According to the univariate logistic regression analysis, several factors were found to be associated with the occurrence of SAP, including age, atrial fibrillation, history of PPI medication, leukocyte, neutrophils, lymphocytes, monocytes, hemoglobin, admission blood glucose, GLR, NIHSS score, gastric tube use, length of hospital stay, and in‐hospital mortality were found to be significantly associated with SAP. Notably, the length of hospital stay and in‐hospital mortality rate were identified as important outcome variables for the occurrence of SAP, with odds ratios (OR) of 1.133 (95% CI: 1.092–1.175, *p* < 0.001) and 5.023 (95% CI: 2.263–11.15, *p* < .001), respectively, indicates that the hospitalization time and in‐hospital mortality rate of SAP group patients are higher than those of Non‐SAP group patients. Variables that demonstrated statistical significance in the univariate analysis and did not overlap in influence with this study were subsequently adjusted for in a multivariate logistic regression analysis. The results revealed that age (OR: 1.027, 95% CI: 1.010–1.043, *p* = 0.001), neutrophil (OR: 1.169, 95% CI: 1.072–1.274, *p* < 0.001), GLR (OR: 1.182, 95% CI: 1.090–1.281, *p* < 0.001), NIHSS score (OR: 1.089, 95% CI: 1.045–1.134, *p* < 0.001), and gastric tube (OR: 6.658, 95% CI: 3.927–11.289, *p* < 0.001) were identified as independent risk factors for the occurrence of SAP (Table 2).

**TABLE 2:** Univariate and multivariate logistic regression analyses.

Variables	Univariate		Multivariate	
	OR (95% CI)	*p* value	OR (95% CI)	*p* value
Age, years	1.027 (1.015–1.040)	<0.001	1.027 (1.010–1.043)	0.001^*^
Atrial fibrillation	2.215 (1.262–3.890)	0.006	0.812 (0.361–1.826)	0.615
PPI medication history	2.214 (1.496–3.276)	<0.001	0.946 (0.594–1.506)	0.814
Leukocyte	1.265 (1.191–1.343)	<0.001	−	−
Neutrophil	1.338 (1.255–1.425)	<0.001	1.169 (1.072–1.274)	<0.001^*^
Lymphocyte	0.314 (0.234–0.422)	<0.001	−	−
Monocyte	4.271 (2.065–8.833)	<0.001	1.555 (0.540–4.477)	0.413
Hemoglobin	0.990 (0.983–0.998)	0.009	0.998 (0.989–1.008)	0.725
Admitted blood glucose (ABG)	1.207 (1.133–1.286)	<0.001	−	−
Glucose‐to‐lymphocyte ratio (GLR)	1.370 (1.277–1.469)	<0.001	1.182 (1.090–1.281)	<0.001^*^
NIHSS score	1.208 (1.168–1.250)	<0.001	1.089 (1.045–1.134)	<0.001^*^
Gastric tube	16.236 (10.493–25.123)	<0.001	6.658 (3.927–11.289)	<0.001^*^
Length of stay, >14 days	1.133 (1.092–1.175)	<0.001	−	−
In‐hospital mortality	5.023 (2.263‐11.150)	<0.001	−	−

### 3.3 Trend Relationship of the GLR Grouping

The GLR was categorized into three groups based on tertiles: the low quantile group Q1 (GLR < 3.05), middle quantile group Q2 (3.05 ≤ GLR < 4.85), and high quantile group Q3 (GLR ≥ 4.85). Group Q1 was used as the reference group to progressively adjust for the remaining covariates to further investigate the relationship between the GLR and the risk of SAP. The results indicated that the risk of SAP in the Q2 group was 2.356 times greater than that in the Q1 group (95% CI: 1.365–4.065, *p* = 0.002), whereas the risk of SAP in the Q3 group was 3.210 times greater than that in the Q1 group (95% CI: 1.782–5.780, *p* < 0.001). The trend test of the three models showed yielded *p* < 0.001, suggesting that there was a gradual increase in the risk of SAP with yielded values of GLR (Table 3).

**TABLE 3:** Trends in multivariable adjusted changes in GLR and stroke‐associated pneumonia.

GLR	Events *N* (%)	Model 1		Model 2		Model 3	
		OR (95% CI)	*p* value	OR (95% CI)	*p* value	OR (95% CI)	*p* value
Q1 (GLR <3.05)	33 (14.0)	Ref.	−	Ref.	−	Ref.	−
Q2 (3.05 ≤ GLR <4.85)	76 (31.9)	2.886 (1.826–4.561)	<0.001	2.170 (1.320–3.568)	0.002	2.356 (1.365–4.065)	0.002
Q3 (GLR ≥ 4.85)	128 (54.0)	7.224 (4.616–11.305)	<0.001	3.904 (2.375–6.417)	<0.001	3.210 (1.782–5.780)	<0.001
*p* for trend		<0.001		<0.001		<0.001	

*Note*: **Model 1**: uncorrected; **Model 2**: corrected for age, gender, NIHSS; **Model 3**: corrected for age, gender, NIHSS, hypertension, stroke history, hyperlipidemia, coronary heart disease, atrial fibrillation, diabetes, smoking, alcohol drinking, PPI medication history, neutrophil, monocyte, erythrocyte, platelet, gastric tube.

### 3.4 Predictive Efficiency Analysis

The area under the curve (AUC) of the GLR was 0.737 (95% CI: 0.698–0.775, *p* < 0.001), when the cutoff value was set at 4.110, the sensitivity was 70.0% and the specificity was 67.1% (Table 4). In paired comparisons with lymphocyte and admission blood glucose (two components of GLR) (*p* < 0.0167 after Bonferroni correction is considered statistically significant), there is a statistically significant difference in AUC values between GLR and lymphocyte, with an observed difference of 0.037 (*p* = 0.011). Similarly, there was a significant difference in AUC between GLR and admission blood glucose (ABG), with an observed difference of 0.068 (*p* = 0.001). When comparing lymphocyte and ABG, there was no significant difference in AUC values (*p* = 0.325). The AUC value indicates that GLR is superior to lymphocyte or ABG in terms of predictive efficiency: GLR (0.737) > lymphocyte (0.699) and GLR (0.737) > ABG (0.669).

**TABLE 4:** Predictive efficiency of GLR and related indicators for the occurrence of SAP.

Variables	AUC (95% CI)	Sensitivity	Specificity	Optimal cutoff value	*p* value
Lymphocyte (LYM)	0.699 (0.657–0.741)	57.8%	75.3%	0.738	<0.001
Admitted blood glucose (ABG)	0.669 (0.627–0.711)	73.4%	55.9%	5.505	<0.001
Glucose‐to‐lymphocyte ratio (GLR)	0.737 (0.698–0.775)	70.0%	67.1%	4.110	<0.001
Neutrophil‐to‐lymphocyte ratio (NLR)	0.745 (0.704–0.785)	63.3%	75.3%	3.966	<0.001
Systemic inflammatory response index (SIRI)	0.735 (0.694–0.776)	57.0%	80.0%	2.325	<0.001

**FIGURE 2:**
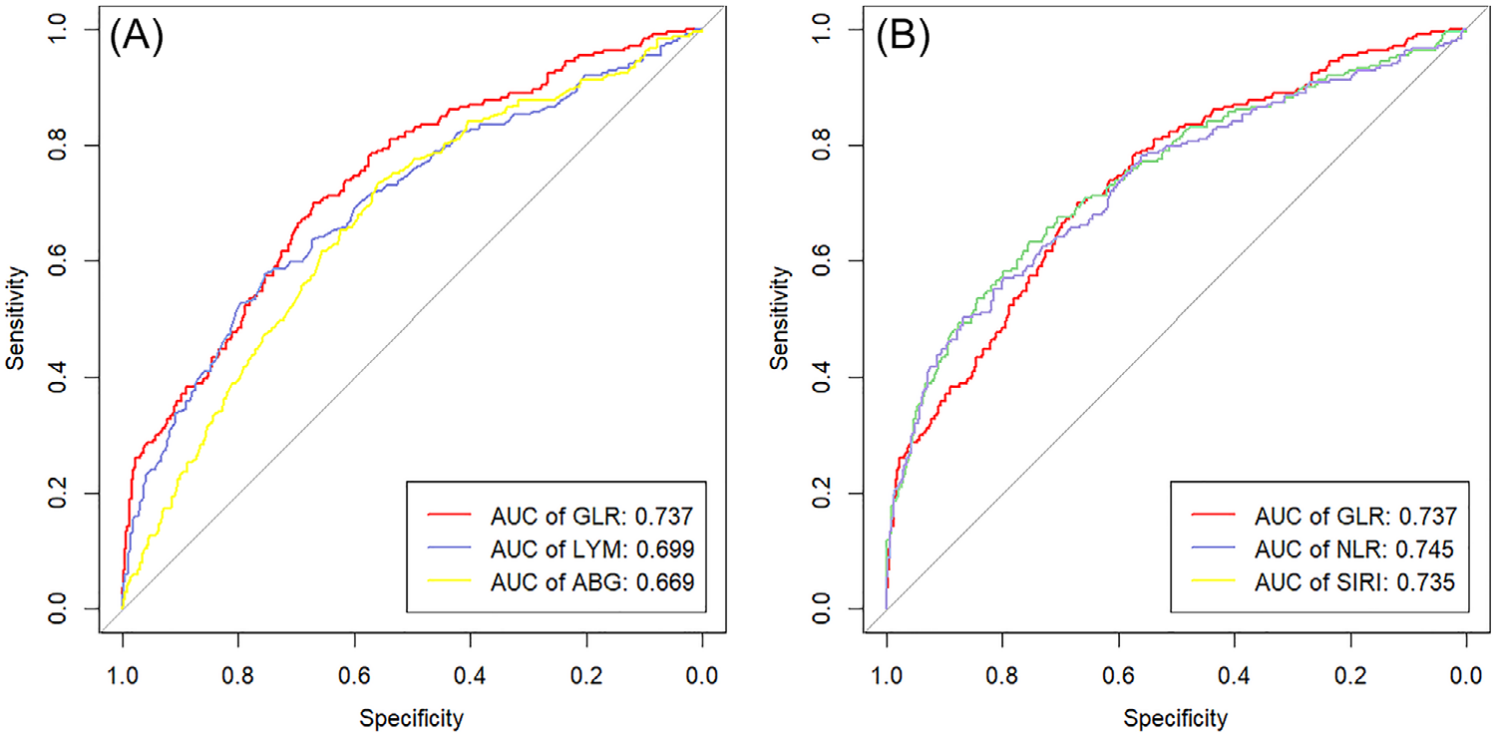
**(A)** Comparison of AUC between GLR and composition indicators LYM and ABG; **(B)** Comparison of AUC between GLR and similar indicators NLR and SIRI. ABG, admitted blood glucose; GLR, glucose‐to‐lymphocyte ratio; LYM, lymphocyte; NLR: neutrophil‐to‐lymphocyte ratio; SIRI: systemic inflammatory response index.

Based on this data, in the comparison of GLR with similar indicators NLR and SIRI, the three groups showed very small differences in AUC, and no significant differences were found in their AUC pairing comparison at the significance level after Bonferroni correction (*p* > 0.0167). This result suggests that GLR is at a similar level of predictive power to NLR and SIRI, and both have a certain degree of accuracy and effectiveness in the early prediction of SAP. However, in terms of the detection sensitivity of their respective optimal cutoff values, GLR has better performance, which has better clinical practicality for using this clinically accessible indicator to take early preventive measures against the occurrence of SAP (Figure 2, Table 4).

## 4 Discussion

This study revealed that an elevated GLR within 24 h after acute stroke admission was an independent risk factor for SAP. The trend test revealed a progressive increase in the risk of SAP with increasing GLR, suggesting that the GLR may have early predictive value for the occurrence of SAP.

The concept of SAP was initially proposed by Hilker in 2003, and represents a significant risk factor for poor prognosis among stroke patients (Hilker et al. 2003). Clinically, the early manifestations of SAP are often atypical, which can result in missed opportunities for timely intervention, leading to an extended length of hospital stay, increased mortality rates, and increased medical expenses (Wilson 2012). In our study, elderly patients, higher NIHSS score, and gastric tube use were more likely to develop SAP, which consequently led to extended hospital stays and Increased in‐hospital mortality rate, these findings are consistent with previous studies' results (Ji et al. 2013; Nam et al. 2018; Cao et al. 2021). Current research has demonstrated a close association between high blood glucose levels and lymphocyte reduction upon stroke admission with the occurrence of SAP (Zonneveld et al. 2017; Hoffmann et al. 2017). On the basis of this evidence, we hypothesize that the GLR, which is composed of these two factors, can provide a more comprehensive and objective reflection of the body's immune status during stroke onset, and may also serve as a predictive indicator for SAP occurrence. Previous studies conducted by Yang et al. and Zhang et al. focused on GLR within fields related to stroke mortality and AECOPD, respectively, and provided theoretical support for our hypothesis. To our knowledge, this is the first study to investigate the relationship between the GLR and SAP.

Research conducted by Zonneveld et al. in 2017 demonstrated a significant correlation between elevated blood glucose levels at the time of admission and the incidence of poststroke infections, particularly pneumonia. In nondiabetic stroke patients, higher blood glucose levels at admission are positively associated with poor functional outcomes and increased mortality within 3 months following stroke (Zonneveld et al. 2017). A study by Elhefnawy et al. in 2024 revealed that patients who experienced a stroke with elevated blood glucose presented a greater incidence of SAP than those with normal blood glucose levels (Elhefnawy et al. 2024). In this study, compared with that in the Non‐SAP group, the OR of admission blood glucose in the SAP group was 1.207, suggesting that hyperglycemia is a risk factor for SAP, which is consistent with the previous results of Zonneveld et al. and Elhefnawy et al., further confirming the association between hyperglycemia and SAP. The current literature suggests several mechanisms through which hyperglycemia may increase the risk of SAP. First, hyperglycemia induces endothelial dysfunction in cerebral and pulmonary small vessels, leading to reduced release of nitric oxide (NO), impaired endothelial repair, and inadequate blood oxygen supply, resulting in chronic inflammatory vascular injury and atherosclerosis (Edgar et al. 2021; Shi and Vanhoutte 2017). Second, hyperglycemia can upregulate the expression of toll‐like receptors, thereby inhibiting neutrophil function. Additionally, elevated blood glucose levels can result in the glycosylation of immunoglobulins and increased oxidative stress, leading to compromised and dysregulated immune responses (Jafar et al. 2016). During stroke onset, whether due to poorly controlled diabetes, stress‐induced hyperglycemia, or other factors such as dietary influences, elevated blood glucose levels can result in a relatively suppressed immune function, thereby increasing susceptibility to infections. Furthermore, a study by Nobs et al. in 2023 revealed that high blood glucose levels impair the activation of lung dendritic cells (DCs) and T cells in diabetic patients, contributing to increased vulnerability to pneumonia due to defects in adaptive immune responses (Nobs et al. 2023).

Stroke‐induced immunodepression (SIID) is one of the main causes of SAP. A study conducted by Feng et al. in 2018 demonstrated that acute stroke can lead to alterations in lymphocyte count, increasing the incidence of infection events, and peripheral blood lymphocytes in the early stage of stroke can help to predict the risk of SAP (Feng et al. 2018). In this study, compared with that in the Non‐SAP group, the OR of lymphocyte in the SAP group was 0.314, suggesting that low lymphocytes are a risk factor for SAP and that the change in lymphocyte count caused by immunosuppression after stroke is related to the occurrence of SAP. Research by Hoffmann et al. in 2017 highlighted that the immune suppression resulting from stroke is mediated by the activation of the sympathetic and parasympathetic nervous systems, as well as the hypothalamic‐pituitary‐adrenal (HPA) axis. This immune suppression is characterized by diminished immune cell functionality, reduced peripheral blood lymphocyte counts, and increased apoptosis (Hoffmann et al. 2017). Moreover, findings from Shim et al. in 2016 indicated that poststroke brain tissue releases immune regulatory mediators, including interleukin‐1β (IL‐1β), tumor necrosis factor‐α (TNF‐α), and calcitonin gene‐related peptide, which attenuate the inflammatory response. This process leads to a decrease in the immune function of neutrophils, natural killer (NK) cells, and T helper 1 (Th1) cells, culminating in systemic immune suppression (Shim and Wong 2016). Concurrently, damage to brain tissue and disruption of the blood–brain barrier (BBB) increase the likelihood of pathogen invasion, thereby increasing the susceptibility of stroke patients to SAP.

The aforementioned studies demonstrated a strong association between hyperglycemia, poststroke immunodepression, and the development of SAP. These findings suggest that the combined inhibition of immune function resulting from these two factors may serve as a crucial mechanism for SAP. Consequently, we speculated that the GLR, which integrates blood glucose and lymphocyte levels upon admission, can provide a more comprehensive and objective reflection of the body's immune status following stroke. In 2023, Yang et al. investigated the correlation between the GLR and all‐cause mortality in stroke patients that there is a linear relationship between increasing GLR and increased mortality during hospitalization (Yang et al. 2023). Similarly, Zhang et al. in 2022 identified the GLR as an independent predictor of in‐hospital mortality in patients with AECOPD, exhibiting predictive capabilities that surpassed those of other inflammatory biomarkers, such as the NLR and the platelet‐to‐lymphocyte ratio (PLR) (Hu et al. 2022). Furthermore, GLR has also been reported in research on ARDS, AMI, and tumors (Zhang and Zhang 2022; Liu and Hu 2023; Yang et al. 2022), but there is currently no research on the application of GLR in SAP. Our findings indicate that the GLR serves as an independent risk factor for the occurrence of SAP. When GLR was categorized into distinct groups, group Q1 (GLR < 3.05) was used as the reference group, and the risk of SAP was 2.356 times greater in group Q2 (3.05 ≤ GLR < 4.85) than in group Q1 and 3.210 times greater in group Q3 (GLR ≥ 4.85) than in the group Q1. The trend test demonstrated a gradual increase in SAP risk with increasing levels of GLR. ROC curve analysis and comparison of predictive efficiency revealed that the AUC for the GLR was 0.737, with a sensitivity of 70.0% and specificity of 67.1% at a cutoff value of 4.110. The predictive efficiency of the GLR for SAP was superior to that of single blood glucose or lymphocyte: the GLR (0.737) > lymphocyte (0.699) and GLR (0.737) > ABG (0.669), and the differences in the AUC were statistically significant (*p* < 0.0167). These results align with our initial hypothesis, suggesting that the GLR may possess a certain early predictive value for SAP. Moreover, the GLR provides a more comprehensive reflection of the body's immune level during stroke than most similar indicators do. Current predictive indicators for SAP, such as the NLR and SIRI, rely solely on routine blood parameters, thereby neglecting the critical influence of blood glucose on the overall immune status. The impact of blood glucose on SAP is manifested primarily through inflammatory damage to small blood vessels in the brain and lungs, as well as through systemic immune suppression. Blood glucose levels during a stroke can serve as a reflection of the current immune status and may indicate potential trends in subsequent immune function alterations. By integrating blood glucose levels with lymphocyte counts, the GLR provides a more holistic representation of the body's overall immune level. Among the three indicators of GLR, NLR, and SIRI established based on these data, the sensitivity and specificity of GLR remain at a good level, while NLR and SIRI, although having excellent specificity, seemed to have lower sensitivity in detection than GLR. In clinical applications, two indicators with different characteristics can be used to complement each other's advantages. These easily obtainable indicators in clinical practice have good clinical practicality for taking early preventive measures against the occurrence of SAP.

This study has several limitations. First, this was a single‐center retrospective analysis. Despite multiple trials and validations of the study design and statistical analyses, the predictive efficiency of the GLR may fluctuate to a certain extent due to the inherent limitations of a single‐center sample. Furthermore, the data collection time included the COVID‐19 epidemic period, the COVID‐19 infection that occurred in the hospitalization period may have an impact on the basic level of lymphocytes, which may be a common confusion factor in studies involving the epidemic period of COVID‐19. Finally, additional laboratory indicators such as high‐sensitivity C‐reactive protein (hs‐CRP) and glycated hemoglobin (HbA1c) were not included in the analysis, which may introduce potential biases. Future research can further analyze the predictive value of GLR in different stroke subtypes, conduct prospective cohort studies and nested case–control studies, develop predictive models, and validate them with external datasets. These efforts will further confirm the diagnostic value and practicality of GLR in clinical practice.

## 5 Conclusions

An elevated GLR within 24 h of hospital admission following an acute stroke is an independent risk factor for SAP. The risk of SAP increases progressively with increasing GLR, suggesting that the GLR may have a certain early predictive value for the occurrence of SAP.

## Author Contributions

**Fuqiang Zhou**: conceptualization, methodology, formal analysis, writing – original draft, writing – review and editing. **Haimei Sun**: project administration, writing – review and editing, supervision, resources. **Liju Ma**: investigation, resources. **Haijiang Li**: methodology, writing‐review and editing. **Min Li**: investigation. **Heying Yang**: methodology. **Ye Xu**: visualization. **Fengchen Gao**: writing‐original draft. **Kuankuan Dang**: data curation.

## Conflicts of Interest

The authors declare that there are no conflicts of interest associated with this study.

## Ethics Statement

This study was approved by the Ethics Committee of the First Affiliated Hospital of Kunming Medical University [Ethics Review L No. 11 (2022)]. The need for informed consent was waived by the Institutional Review Board because all personal identifiers in the retrospective study were removed beforehand. This study complies with the ethical principles of the Declaration of Helsinki.

### Peer Review

The peer review history for this article is available at https://publons.com/publon/10.1002/brb3.70404.

## Data Availability

The data that support the findings of this study are available from the corresponding author upon reasonable request.
